# Clinical and radiological results of reverse total shoulder arthroplasty with or without lateralization as revision procedure for failed arthroplasty

**DOI:** 10.1016/j.jseint.2024.10.014

**Published:** 2024-11-27

**Authors:** Jan-Philipp Imiolczyk, Laurent Audigé, Florian Freislederer, Philipp Moroder, David Endell, Raphael Trefzer, Markus Scheibel

**Affiliations:** a Department of Shoulder and Elbow Surgery, Center for Musculoskeletal Surgery, Charité Universitaetsmedizin, Berlin, Berlin, Germany; b Research and Development, Schulthess Clinic, Zürich, Switzerland; c Surgical Outcome Research Center, Department of Clinical Research, University Hospital Basel and University of Basel, Basel, Switzerland; d Shoulder and Elbow Surgery, Schulthess Clinic, Zürich, Switzerland

**Keywords:** Metallic augmentation, Secondary, Aseptic, Cuff failure, Glenoid defects, Complication, Bipolar, Loosening

## Abstract

**Background:**

As reverse total shoulder arthroplasty (rTSA) becomes a common treatment option in the revision setting, common problems associated with Grammont’s design such as scapular notching, instability, and rotator cuff weakening occur. Design changes associated with superior outcomes in primary rTSA, such as glenoid or humeral lateralization have not yet been examined in the revision settings. The purpose of this consecutive series of revision rTSA is to evaluate the clinical and radiological short-term results after aseptic and septic revision rTSA and explore potential benefits of metallic glenoid and humerus lateralization.

**Methods:**

In this study, patients treated with an rTSA between 2014 and 2020 after failed shoulder arthroplasty were included. Forty-five consecutive patients were divided into comparative groups using lateralized rTSA with metallic baseplate augmentation (latrTSA) and additional humeral lateralization using a 145° onlay curved stem (bi-latrTSA); or no baseplate offset with a Grammont-type 155° stem (non-latrTSA). Further, outcome of postinfection revision rTSAs was compared to aseptic loosening. Constant-Murley-Score, subjective shoulder value, shoulder range of motion including Apley’s scratch test, abduction strength, and pain levels were assessed. Radiographs were reviewed for implant loosening, scapular notching, fractures, and osteolysis. Lateralization and distalization shoulder angle were measured at the final follow-up.

**Results:**

Thirty-eight patients showed significant improvement in all functional measurements at the final 2-year follow-up compared to the baseline (*P* < .01). There were no significant differences in favor of glenoid or bipolar lateralization. However, no scapular notching was seen in patients with both humeral and glenoid lateraliazion (non-latrTSA: 33%; latrTSA: 8%; bi-latrTSA: 0%; *P* = .103), with no signs of implant loosening. Patients with bi-latrTSA showed significantly greater lateralization shoulder angle (*P* = .017); distalization shoulder angle was lower, but not significantly (*P* = .230). Postinfectious rTSA after aseptic loosening (n = 19; 55%) presented better internal rotation (*P* = .036) compared to postinfectious rTSA. The overall complication rate was 16% and 8% leading to revision.

**Conclusion:**

rTSA is a viable option for revision cases and presents good results after failed shoulder arthroplasty, including the infected shoulder. The effect of metallic augmentation on clinical results is not comparable to those in literature in primary rTSA setting due to advanced preoperative medialization. However, scapular notching was prevented in all cases with bipolar lateralization.

As the incidence of shoulder arthroplasties is on the rise,^31^ the number of failed prostheses in need of revision continues to steadily increase. This trend has been recently captured by Gauci et al, who reported a reintervention rate of 12.6% from 1993 to 2013 in Europe.^17^ Indications for reverse total shoulder arthroplasty (rTSA) as a revision procedure include severe glenoid bone loss,^25^ secondary rotator cuff dysfunction,^28^ persistent pain,^27^ subscapularis failure in anatomic arthroplasties,^15^ with glenoid instability and infection as the most frequently causes requiring multiple revisions.^17^

In primary arthroplasty, the medialized Grammont-style design has presented some disadvantages including scapular notching, poor improvement in active rotation, instability, glenoid loosening or implant breakage, and loss of the natural shoulder contour.^1^^,^^4^^,^^5^^,^^10^ To overcome these limitations, lateralization at the glenoid, the humerus or on both sides (i.e. bipolar lateralization) is used to increase deltoid recruitment (of anterior and posterior muscle fibers)^7^ and improve balance of the remaining forced couples ^19^ to gain range of motion (ROM).^3^^,^^47^, ^48^, ^49^ New designs embrace the possibility of metallic augmentation, which is particularly attractive in revision settings, where bone stock is limited. Clinical effects of metallic augmentation in revision cases have not yet been investigated. The aim of this study was to evaluate the clinical and radiological results of a consecutive patient cohort of revision rTSA and to explore effects of metallic augmentation in this cohort.

## Materials and methods

### Study population

Patients who underwent rTSA between January 2014 and December 2020, in one of our 2 institutions, were screened. rTSA performed as a revision procedure of prior ipsilateral (anatomical hemi or total, or reverse) shoulder arthroplasty surgery were included. Patients who were treated with a primary rTSA (i.e. cuff-tear arthropathy, osteoarthritis), acute fractures, tumor, or revision procedures after osteosynthesis (fracture sequalae) were excluded. Further exclusion criteria were severe humeral bone loss that required additional allograft or autograft composite. All patients were documented prospectively and followed up at 12 and at least 24 months after implantation.

For subgroup analysis, patients were split into 3 groups based on their surgical treatment; 2 groups were implanted a lateralized-rTSA design using either exclusively glenoid augmentation (latrTSA) or both humeral and glenoid lateralization (bi-latrTSA) compared to a patient group treated with a medialized Grammont-style rTSA design (non-latrTSA). Depending on the primary cause for revision (i.e. presence of infection at presentation), patients were divided in 2 groups (septic vs. aseptic group).

### Implant design

On the glenoid side, all patients were either treated with Aequalis Reversed II or Tornier Perform Reversed Glenoid (Stryker Corp., Kalamazoo, MI, USA). Based on the chronological implantation, until June 2016, patients with Reversed II did not receive any metallic augmentation on the glenoid side at one center. At the second center, metallic augmentation was used to surgeons’ individual preference.

The glenoid components of the Perform Reversed system included a porous titanium baseplate (25- or 29-mm diameter) designed to encourage bone ingrowth^35^ with an additional central screw up to 4 peripheral screws used for glenoid fixation. Metallic augmentation of the baseplate was performed using +3-mm or +6-mm offset. The amount of offset was determined by the surgeon’s discretion individually for each patient intraoperatively.

On the humerus, 2 different implants were used based on the quality and quantity of metaphyseal bone stock available. Wherever possible, since its availability, the short curved Tornier Flex stem (Stryker Corp., Kalamazoo, MI, USA) with 145° neck-shaft angle was implanted in combination with glenoid lateralization, where sufficient metaphyseal bone stock was available (bi-latrTSA). According to Werthel et al, this onlay stem design results in additional (humeral) lateralization of 6.2 mm in theory^50^ compared to the otherwise used 155° neck-shaft angle Aequalis Reverse II stem.

### Surgical protocol

All surgeries were performed by 2 senior surgeons (M.S. and P.M.). All patients were placed in a beach-chair position and treated with general anesthesia combined with an interscalene block. All procedures were performed through a standard deltopectoral approach according to the manufacturer’s technical manuals. The size of glenosphere (36, 39, or 42 mm) was determined based on the size of the glenoid. A subscapularis repair was attempted whenever possible.

In cases where radiological, anamnestic, or clinical signs of infection were present, surgery was preceded by a presurgical joint aspiration or arthroscopic lavage. A septic revision was determined by either a positive joint aspiration or at least 3 out 5 positive probes (taken during arthroscopic lavage or during revision rTSA surgery) stating more than 10^5^ pathogens.

A single-stage procedure followed by a 6- to 12-week antibiotic treatment was performed in cases where the pathogen responsible for the infection was identified. In remaining cases, a 2-stage approach was followed: in a first procedure, all material was explanted, a radical débridement was performed, and a probe was collected before the implantation of an antibiotic-loaded cement spacer on the humeral side to locally eradicate the infection. A systemic antibiotic treatment (based on antibiogram, if available) was prescribed to all patients for an additional 6 weeks. After this period, a second surgery was performed to explant the spacer and implant the new prostheses. All patients received a similar antibiotic regime in the 6 following weeks following rTSA implantation. In cases with no signs of infection before revision surgery, the antibiotic regime (started empirically after surgery) was stopped when all intraoperative probes tested negative for pathogens.

### Postoperative rehabilitation

Patients followed a standardized postoperative rehabilitation protocol. The shoulder was immobilized in a sling in internal rotation for 6 weeks. During this time, only passive movement (excluding external rotation above 0°) was allowed during the physiotherapy sessions and slowly increased under supervision. After 6 weeks, active motion was added to the protocol with strengthening exercises commencing after 12 weeks

### Clinical evaluation

All patients were clinically evaluated before surgery (baseline) and at 12 and at least 24 months postoperatively. All data were prospectively collected and recorded in local shoulder arthroplasty registries.^34^

At baseline, parameters including surgical details, patients’ demographic data, cause for revision, and shoulder functionality were recorded. Clinical outcomes were recorded at the baseline and each follow-up (FU) examination using the Constant-Murley Score (CS),^11^ including its pain subscale (0 = no pain; 15 = excruciating pain), patient-reported activity levels, ROM, and abduction strength as well as subjective shoulder value (SSV).^13^ ROM was documented for anterior forward flexion, abduction, and external rotation. Internal rotation was reported by Apley scratch test’s categories. Intraoperative and postoperative complications were documented throughout the entire FU period up to 24 months after surgery.

### Preoperative imaging and radiographic FU

Standardized true anteroposterior, axial, and Y-view radiographs were taken preoperatively and at the 12- and 24-month FU. Preoperative radiographs were used to establish causes for persistent pain or poor function (e.g., implant breakage or loosening, superior head migration as sign of secondary cuff deficiency). Postoperatively, signs of scapular notching (according to Sirveaux classification^45^), implant loosening, osteolysis, radiolucent lines, heterotopic ossification, greater tuberosity resorption and fractures were also reviewed on all available postoperative radiographs.^14^ Furthermore, lateralization (LSA) and the distalization shoulder angle (DSA) were measured.^9^

### Data management and statistical analysis

Registry data were managed using the REDCap Electronic Data Capture system^24^ (Vanderbilt University, Nashville, TN, USA) and exported for statistical analysis using Intercooled Stata version 17 (StataCorp LP, College Station, TX, USA). Patient demographic, radiological, and functional parameters (ROM, strength, SSV, and CS) were tabulated for the entire patient cohort and in addition separately per group at baseline and at 24-month FU using standard descriptive statistics. Clinical parameter changes from baseline to the 24-month FU were analyzed by Wilcoxon signed-rank test and symmetry test for continuous and categorical outcomes, respectively. Comparative analyses at 24 months were conducted using generalized linear mixed models to account for repeated measurements at each clinical FU time point as applicable, followed by standard linear regression analyses. For all models, age at implantation, sex, and preoperative values were used for adjusting group postoperative differences. Regarding scapular notching, only patients were included in the analysis with no notching at baseline. All eligible patients from our joined local registries were included; therefore, no sample size was predetermined at the beginning of the study. All analyses were explorative with a significance level set at 0.05.

## Results

### Patient selection and demographics

**Figure 1:**
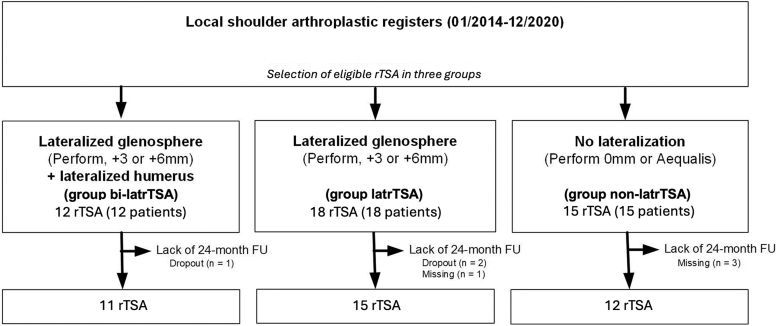
Flowchart of patient selection and follow-up. *Group non-latrTSA*, reverse total shoulder arthroplasty with no baseplate offset with a Grammont-type 155° stem; *Group latrTSA*, lateralized reverse total shoulder arthroplasty with metallic baseplate augmentation; *Group bi-latrTSA*, lateralized reverse total shoulder arthroplasty with metallic baseplate augmentation and additional humeral lateralization using a 145° onlay curved stem.

Between January 2014 and December 2020, 45 revision shoulder arthroplasties were implanted in 45 patients (Fig. 1). Based on a completed 24-month FU examination, the final analysis population included a total of 38 patients, with 18 women (47%), a mean age of 68 (range: 53-83) years old. Patients were divided into 3 subgroups, depending on glenoid or bipolar augmentation. Minor differences between those 3 groups are visible regarding age, sex, and baseline function (Table I). The most frequent causes for revision surgery were infection (45%), followed by glenoid loosening (21%) and rotator cuff insufficiency (18%) (Table II). Based on patients’ individual anatomy and surgeon’s preference, implant configurations were heterogenous throughout the patient cohort regarding eccentricity, glenosphere size, and offset (Table III).

**Table I:** Baseline demographics, shoulder range of motion parameters, strength, and functional scores.

Baseline parameters	Non-latrTSA (1)		latrTSA (2)		Bi-latrTSA (3)		Standardized difference		
	n (%)	mean (SD)	n (%)	mean (SD)	n (%)	mean (SD)	1 vs. 3	1 vs. 2	2 vs. 3
Age at surgery	12	67 (10)	15	71 (6)	11	66 (8)	0.184	0.375	0.681
Sex							0.091	0.067	0.024
Female	6 (50)		7 (47)		5 (45)				
Male	6 (50)		8 (53)		6 (55)				
Flexion (°)	12	50 (31)	15	59 (38)	11	71 (37)	0.626	0.260	0.329
Abduction (°)	12	49 (29)	15	52 (36)	11	73 (31)	0.809	0.109	0.615
External rotation in 0° abd. (°)	12	17 (26)	15	11 (16)	11	22 (20)	0.349	0.189	0.726
Internal rotation (Apley's test)							1.296	1.165	0.646
Lateral thigh	5 (42)		6 (40)		2 (18)				
Buttock	1 (8)		5 (33)		4 (36)				
Lumbosacral region	2 (17)		4 (27)		4 (36)				
Waist (L3)	3 (25)								
T12 vertebra	1 (8)				1 (9)				
Strength in abduction (kg)	12	0.9 (2.1)	15	0.9 (2.4)	11	0.6 (1.1)	0.140	0.055	0.123
Subjective Shoulder Value (%)	12	25 (13)	15	23 (23)	11	27 (20)	0.111	0.112	0.184
Pain level NRS (0-10 = max)	12	6 (3)	15	7 (3)	11	5 (3)	0.195	0.349	0.579
Constant-Murley Score (0 = min 100 = max)	12	23 (16)	15	21 (20)	11	28 (15)	0.336	0.097	0.397

*SD*, standard deviation; *NRS*, Numeric Rating Scale; *Group non-latrTSA*, reverse total shoulder arthroplasty with no baseplate offset with a Grammont-type 155° stem; *Group latrTSA*, lateralized reverse total shoulder arthroplasty with metallic baseplate augmentation; *Group bi-latrTSA*, lateralized reverse total shoulder arthroplasty with metallic baseplate augmentation and additional humeral lateralization using a 145° onlay curved stem; *StdDiff*, standardized difference calculated to 2 decimal places and equal to the absolute difference between group means divided by the common standard deviation, where values closest to 0.10 or below indicate stronger group similarity.

**Table II:** Reasons for revision by reverse total shoulder arthroplasty.

Reasons for revision (combinations are possible)	Non-latrTSA (n = 12)	latrTSA (n = 15)	Bi-latrTSA (n = 11)
	n (%)	n (%)	n (%)
Infection	4 (33)	9 (60)	4 (36)
Periprosthetic fracture (glenoidal/humeral)	-	1 (7)	-
Instability	2 (17)	1 (7)	-
Glenoid component loosening	4 (33)	4 (27)	3 (27)
Humeral component loosening	1 (8)	-	-
Rotator cuff problem	3 (25)	2 (13)	5 (45)
Prothesis failure	1 (8)	2 (13)	1 (9)
Other reasons∗	1 (8)	1 (7)	1 (9)

*Group non-latrTSA*, reverse total shoulder arthroplasty with no baseplate offset with a Grammont-type 155° stem; *Group latrTSA*, lateralized reverse total shoulder arthroplasty with metallic baseplate augmentation; *Group bi-latrTSA*, lateralized reverse total shoulder arthroplasty with metallic baseplate augmentation and additional humeral lateralization using a 145° onlay curved stem.

∗ Other reasons include: non-latrTSA, Progressive omarthrosis with Hemi-TP. latrTSA, Tuberculosis dislocation and secondary RM, insufficiency. bi-latrTSA, Secondary glenoid erosion.

**Table III:** Reverse total shoulder arthroplasty implant configurations and operative details.

Implant configurations	Non-latrTSA (n = 12)	latrTSA (n = 15)	Bi-latrTSA (n = 11)
	n (%)	n (%)	n (%)
Endoprosthesis type			
Aequalis™ Reversed II	12 (100)	15 (100)	
Tornier Perform™ Reversed Glenoid			11 (100)
Baseplate type			
Reversed II standard	9 (75)		
Perform standard	3 (25)	15 (100)	11 (100)
Baseplate offset			
Standard	12 (100)		
Lateralized +3		9 (60)	10 (91)
Lateralized +6		6 (40)	1 (9)
Glenosphere size			
36 mm	11 (92)	13 (87)	8 (73)
39 mm			1 (9)
42 mm	1 (8)	2 (13)	2 (18)
Glenosphere type			
Centered	7 (58)	3 (20)	
Eccentric	5 (42)	12 (80)	11 (100)

*Group non-latrTSA* reverse total shoulder arthroplasty (rTSA) with no baseplate offset with a Grammont-type 155° stem; *Group latrTSA*, lateralized rTSA, with metallic baseplate augmentation; *Group bi-latrTSA*, lateralized rTSA, with metallic baseplate augmentation and additional humeral lateralization using a 145° onlay curved stem.

### Clinical and radiological outcome

Patients presented clinical results with a mean CS of 61 (standard deviation [SD]: 17) points and SSV of 67% (SD: 20) at 24 months. Our patients presented an improvement in CS from baseline of 37 (95% confidence interval (CI): 31-43) points and of 42% (95% CI: 36-49) in SSV (*P* < .001). There was a statistically significant increase in all outcome measurements compared to the baseline (*P* < .010) (Table IV). Levels of pain decreased to 1.9 (SD: 2.2) points on average with a final abduction strength of 3.7 (SD: 2.9) kilograms and a mean increase in external rotation of 11° (95% CI: 5-17), 75° of flexion (95% CI: 62-88) and 68° of abduction (95% CI: 56-81) after revision rTSA. Scapular notching rate was 14.7% (grade 1: n = 2; grade 2: n = 3).

**Table IV:** Change of shoulder range of motion parameters, strength, and functional scores.

Outcome parameters	Baseline		Final FU		Change (95% CI)	*P* value
	n (%)	mean (SD)	n (%)	mean (SD)		
Flexion (°)	38	60 (36)	36	134 (34)	75 (62-88)	<.001
Abduction (°)	38	57 (34)	36	126 (37)	68 (56-81)	<.001
External rotation in 0° abduction (°)	38	16 (21)	34	28 (22)	11 (5-17)	.001
Internal rotation (Apley's test)						.006
Lateral thigh	13 (34)		3 (9)			
Buttock	10 (26)		12 (34)			
Lumbosacral region	10 (26)		9 (26)			
Waist (L3)	3 (8)		8 (23)			
T12 vertebra	2 (5)		1 (3)			
Interscapular T7			2 (6)			
Strength in abduction (kg)	38	0.8 (2.0)	36	3.7 (2.9)	2.9 (2.0-3.7)	<.001
Subjective Shoulder Value (%)	38	25 (19)	38	67 (20)	42 (36-49)	<.001
Pain level NRS (0-10 = max)	38	6.1 (3.0)	38	1.9 (2.2)	−4.2 (−5.1 to −3.3)	<.001
Constant-Murley Score (0 = min 100 = max)	38	24 (17)	35	61 (17)	37 (31-43)	<.001

*SD*, standard deviation; *CI*, confidence interval; *NRS*, Numeric Rating Scale.

*P* value = Wilcoxon signed-rank test *P* value (symmetry test however was used for the Apley's test ordered categorical data).

There was no evidence of implant loosening, osteolysis, or periprosthetic fractures or scapular stress fractures. Three patients each presented one radiolucent line around the metaphysis of the stem and one with heterotopic ossification (grade 1) at the final FU. Eleven cases (29%) presented with poor metaphyseal bone stock and complete greater tuberosity resorption, hence no measurements of LSA and DSA were possible in those cases.

### Glenoid and bipolar metallic augmentation

**Figure 2:**
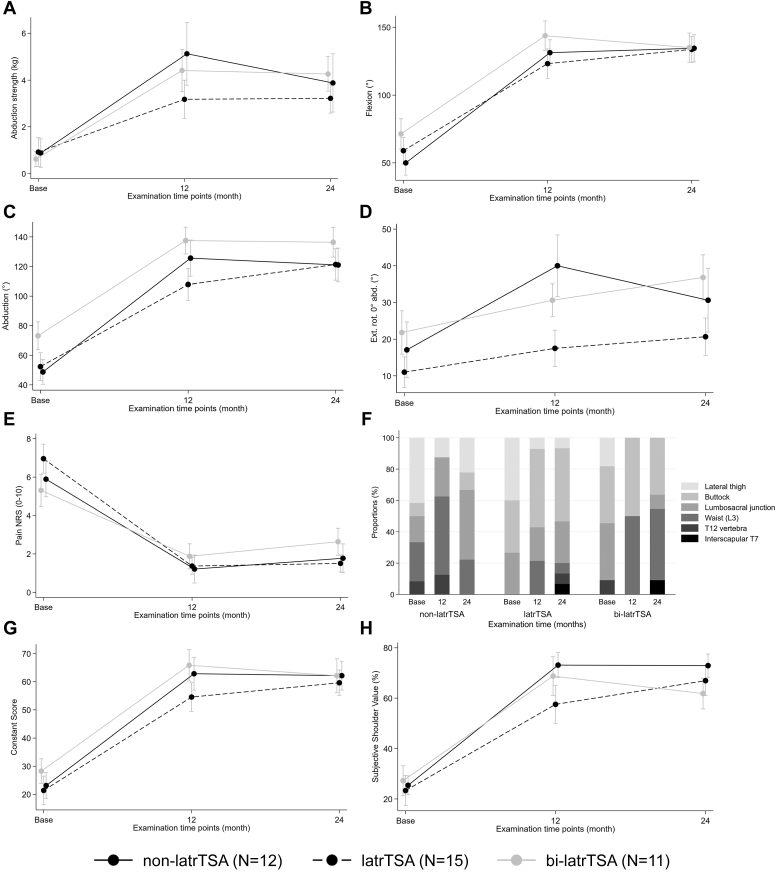
Clinical results. *Group non-latrTSA*, reverse total shoulder arthroplasty with no baseplate offset with a Grammont-type 155° stem; *Group latrTSA*, lateralized reverse total shoulder arthroplasty with metallic baseplate augmentation; *Group bi-latrTSA*, lateralized reverse total shoulder arthroplasty with metallic baseplate augmentation and additional humeral lateralization using a 145° onlay curved stem.

Compared to baseline function, all groups showed an improvement in all functional outcome measurements besides for internal rotation (Fig. 2, Supplement 1). Patients with no augmentation provided no improvement, patients with glenoid augmentation provided some improvement, however not significant, while patients with bipolar augmentation presented a trend towards better internal rotation (Supplement 1 and 2). Regarding external rotation, no lateralization presented with a nonsignificant improvement of 9° (95% CI: −1 to 19; *P* = .103), whereas both glenoid and bipolar lateralization displayed an significant improvement of 10° (95% CI: 2-18; *P* = .026) an 15° (95% CI: 3-27; *P* = .045), respectively.

Scapular notching was present at 24-month FU in 33.3% (grade 1: n = 2; grade 2: n = 2) vs. 8.3% (grade 2: n = 1) vs. 0% (*P* = .103) of patients from the non-latrTSA, latrTSA, and bi-latrTSA groups, respectively. Two cases (lat-rTSA) of grade 1 notching were present at baseline after periprosthetic infection of an rTSA and were therefore removed from this analysis; in 2 cases external radiography were provided by the patient where assessment was not possible. Radiologic measurements show significantly greater LSA and trends towards lower DSA in bi-latrTSA patients (Table V); LSA was greater bi-latrTSA patients by 12.5° (95% CI 0.4-24.7°) compared to non-latrTSA (adjusted *P* value = .044).

**Table V:** Radiological measurements.

Radiological angle parameters (°)	Non-latrTSA	Mean (SD)	latrTSA	Mean (SD)	Bi-latrTSA	Mean (SD)	*P* value
	n		n		n		
Lateralization angle	10	71.7 (5.7)	6	68.0 (4.9)	11	84.8 (17.6)	.017
Distalization angle	10	54.5 (9.4)	6	57.9 (10.2)	11	47.6 (15.5)	.230

*SD*, standard deviation; *Group non-latrTSA*, reverse total shoulder arthroplasty with no baseplate offset with a Grammont-type 155° stem; *Group latrTSA*, lateralized reverse total shoulder arthroplasty with metallic baseplate augmentation; *Group bi-latrTSA*, lateralized reverse total shoulder arthroplasty with metallic baseplate augmentation and additional humeral lateralization using a 145° onlay curved stem.

### Aseptic vs. septic revision rTSA

There were 21 patients that were treated with a revision rTSA due to an aseptic cause. Leading causes for revision were glenoid loosening (n = 9), rotator cuff deficiency (n = 6), instability (n = 2), secondary glenoid erosion after hemi arthroplasty (n = 2), or implant failure (n = 2). Those patients presented at the final FU with a CS of 63 (SD: 19) points and an SSV of 70% (SD: 21) regardless of component configuration.

Seventeen patients were treated due to septic loosening or infection of previous implants. Final functional outcome with regards to a CS of 59 (SD: 16) points and an SSV of 64% (SD: 19) was similar (Table VI).

**Table VI:** Outcome comparison between infection and noninfection-related revision using reverse total shoulder arthroplasty.

Outcome parameters	Noninfection		Infection		Change (95% CI)	*P* value
	n (%)	mean (SD)	n (%)	mean (SD)		
24-mo outcome						
Flexion (°)	19	129 (35)	17	140 (33)	11 (−12 to 33)	.270
Abduction (°)	19	127 (35)	17	125 (40)	−2 (−26 to 23)	.987
External rotation in 0° abduction (°)	18	31 (22)	16	25 (21)	−7 (−21 to 8)	.412
Internal rotation (Apley's test) (n,%)						.036
Lateral thigh	-		3 (18)			
Buttock	7 (39)		5 (29)			
Lumbosacral region	4 (22)		5 (29)			
Waist (L3)	7 (39)		1 (6)			
T12 vertebra	-		1 (6)			
Interscapular T7	-		-			
Strength in abduction (kg)	19	4.2 (3.2)	17	3.2 (2.5)	−1.0 (−2.9 to 0.8)	.372
Subjective shoulder value (%)	21	70 (21)	17	64 (19)	−6 (−19 to 7)	.293
Pain level NRS (0-10 = max)	21	2.2 (2.6)	17	1.6 (1.5)	−0.6 (−2.0 to 0.8)	.892
Constant-Murley Score (0 = min 100 = max)	18	63 (19)	17	59 (16)	−3 (−15 to 9)	.458
Outcome change from baseline						
Flexion (°)	19	59 (32)	17	92 (43)	33 (8-58)	.010
Abduction (°)	19	55 (23)	17	83 (47)	28 (4-52)	.035
External rotation in 0° abduction (°)	18	5 (16)	16	18 (16)	13 (2-23)	.029
Strength in abduction (kg)	19	2.7 (2.4)	17	3.1 (2.6)	0.4 (−1.2 to 2.1)	.622
Subjective shoulder value (%)	21	37 (21)	17	49 (17)	11 (−1 to 24)	.111
Pain level NRS (0-10 = max)	21	−3.0 (2.8)	17	−5.7 (2.0)	−2.7 (−4.3 to −1.1)	.003
Constant-Murley Score (0 = min 100 = max)	18	30 (16)	17	45 (15)	15 (5-26)	.012

*SD*, standard deviation; *CI*, confidence interval; *NRS*, Numeric Rating Scale.

*P* value = Two-sample Wilcoxon rank-sum (Mann-Whitney) test.

Patients treated due to aseptic loosening presented significantly greater internal rotation (*P* = .036). However, greater improvement compared to baseline was achieved in patients with postinfectious rTSA in flexion (*P* = .010), abduction (*P* = .035), external rotation (*P* = .029), CS (45 vs. 30 points; *P* = .012), and pain (−5.7 vs. −3.0 points in the CS pain subscale; *P* = .03) (Supplement 3).

### Complication

There were similar complication rates in all 3 cohorts (Table VII): 13% (2 of 15) in the latrTSA group, 18% (2 of 11) in bilat-rTSA group, and 17% (2 of 12) in the non-latrTSA group. There was 1 case of instability (2.6%) that was treated with glenoid lateralization. An overall complication rate of 16% with 8% rate of revision was obtained in our cohort.

**Table VII:** Adverse events and their treatment.

n	Group	Adverse event	Intervention
1	non-latrTSA	Intraoperative dislocation of glenosphere and baseplate with complete disassembly over 2 y	Revision surgery with exchange of inlay, glenosphere and baseplate
2	non-latrTSA	Delayed wound healing at the proximal end between 2 weeks and 3 mo postoperatively	-
3	latrTSA	Intraoperative fracture of greater tuberosity	Additional fiber wire fixation
4	latrTSA	Instability at 2 mo postoperatively	Revision with increase in inlay liner
5	bi-latrTSA	Baseplate loosening after 6 mo	Two-stage revision due to possible persistent infection
6	bi-latrTSA	baseplate loosening and consecutive glenoid fracture after 12 mo	Surgical reintervention rejected by patient

*Group non-latrTSA*, reverse total shoulder arthroplasty with no baseplate offset with a Grammont-type 155° stem; *Group latrTSA*, lateralized reverse total shoulder arthroplasty with metallic baseplate augmentation; *Group bi-latrTSA*, lateralized reverse total shoulder arthroplasty with metallic baseplate augmentation and additional humeral lateralization using a 145° onlay curved stem.

## Discussion

Our data present favorable and comparable clinical and radiological results in a heterogenous patients cohort of patients after failed shoulder arthroplasty. All patients have displayed a statistically significant increase in CS from baseline of 37 (95% CI: 31-43; *P* < .001) points to a total of 61 points and an increase of 42% (95% CI: 36-49) in SSV (*P* < .001) to a total of 67% at 24 months. Complication and revision rates were 16% and 8%, respectively. Metallic glenoid augmentation (8.3% notching for latrTSA) presented a significant reduction in scapular notching compared to the non-laterized rTSA design (33.3%), whereas additional humerus lateralization prevented it in all cases (0.0% notching for bi-latrTSA).

These results echo the functional outcomes reported by previous studies: Melis et al analyzed a cohort of 37 revision procedures to rTSA and found an overall CS improvement of 31 points from baseline function, which patients described in 86% as “satisfying” or “very satisfying.”^36^ Merolla et al described a CS increase from 8.5 points at baseline to 40.7 at 36-month FU in 37 failed hemi shoulder arthroplasty (HA) converted to rTSA.^37^ Our results show that glenoid lateralization improved ROM at 24 months after surgery, with a mean (95% Confidence interval) change from baseline of 75° (53°; 96°) in anterior forward flexion, 69° (45°; 93°) in abduction, and 10° (2°; 18°) in external rotation. This ROM improvement is slightly greater than the one recently described by Hao et al, who analyzed a cohort of 45 revision rTSAs secondary to failed anatomical total shoulder arthroplasty (abduction of 34° [SD: 34] and external rotation of 2° [SD: 27]).^23^

The indication for revision rTSA influences the clinical outcome. A high number of pre-existing operations and persisting chronic infection display risk factors for unfavorable outcome.^20^ However, in our study, all patients regardless of their primary indication for revision surgery (i.e., septic vs. nonseptic loosening) showed a functional postoperative improvement at the 24-month FU compared to their baseline status. Statistically significantly greater internal rotation was achieved in patients with aseptic loosening. Patients with septic revision showed greater reduction in pain relief, greater ROM, and a higher median CS score compared to the baseline status, in whom most had a cemented spacer implanted. We hypothesize that patients with septic loosening showed greater pain levels at the baseline and worse function, resulting in a better net gain of function and subjective shoulder quality after rTSA. Functional results are limited in cases, particularly after multiple operations and damage of the soft tissue envelope.^21^ Multiple surgeries each require subscapularis tenotomies and reattachments that might explain the inferior results for internal rotation.

The rate of instability and function of our patient cohort is superior to patients needing allograft prosthesis composite in combination with possible tendon transfers when sever humeral bone stock is missing.^6^^,^^33^^,^^43^

It is well accepted that conversion of a failed total shoulder arthroplasty, HA, or rTSA to rTSA yields pain relief and improved functional outcomes. However, complication rates are overall high and can compromise the functional results.^2^^,^^8^^,^^44^ We observed a postoperative complication rate of 16% and a revision rate of 8% in all patients and no significant differences with regards to implant configuration. However, the literature presents higher complication and revision rates for patients undergoing medialized rTSA.^23^^,^^44^ A current meta-analysis including 107 studies (5010 revision rTSAs) found postoperative complication and revision rates of 22% (n = 722 of 3474) and 15% (n = 533 of 3474), respectively.^40^ In a systematic review analyzing 925 failed primary HA converted to rTSA a revision rate of 10.7% was reported, mostly due to prosthetic loosening, joint instability, and joint dislocation.^41^

Comparing radiological measurements to a cohort of patients with rTSA after cuff-tear arthropathy, a mean LSA of 78° and 83° was obtained for patients treated with Aequalis reversed and Ascend Flex, respectively, with a DSA of 55° for both implants.^30^ Concerning patients with Ascend Flex (bi-latrTSA), LSA is comparable in our cohort (85°), whereas less distalization is achieved on average (48°). Patients treated with Aequalis reversed (non-latrTSA and latrTSA) show similar DSA (55° and 58°) but achieve less lateralization (72° and 68°), compared to 78° for patients with cuff-tear arthropathy. This emphasizes the hypothesis that revision surgeries result in more medialization.

Scapular notching was significantly lower in patients with metallic glenoid lateralization. Similarly, a meta-analysis comparing the efficacy of both lateralized vs. nonlateralized rTSA in preventing scapular notching in 865 patients undergoing primary rTSA reported a lower rate in patients treated with lateralized rTSA (OR = 0.14).^38^ This could not be reproduced in our cohort. Although scapular notching was reduced with metallic glenoid augmentation, only patients with bilateral lateralization showed no notching at all. A possible explanation for our finding is the high degree of glenoid bone loss observed in patients undergoing revision surgery, as it results in a shorter scapula neck, where simply glenoid lateralization does not suffice.

Our results show that metallic glenoid lateralization is a suitable augmentation in revision cases with good clinical results and reduced scapular notching. The functional outcomes yielded by metallic glenoid lateralization were not superior to the ones observed in patients treated with a medialized concept. Contrary to primary rTSA, in which glenoid lateralization achieves better external rotation in primary setting^22^ than the Grammont-style medialized rTSA,^38^^,^^46^ our study revealed that in our patient cohort, very good results were achieved in all patients, regardless of implant configuration.

Additional humeral lateralization improves stability,^32^ lever arm of the deltoid^26^^,^^18^^,^^12^ and deltoid wrapping,^42^ demonstrating improved clinical function when introduced in combination to bony^16^^,^^39^ or metallic ^29^ glenoid augmentation in primary settings. In our revision cohort, additional humeral lateralization did not impact shoulder function as expected, despite the availability of better humeral metaphyseal bone stock. Twenty-four percent of patients presented complete greater tuberosity resorption, which might also diminish effects of humeral lateralization.

There are several limitations to this study. First, this was a retrospective study with all outcome measures prospectively collected in a clinical database. Second, the study population size was small and heterogeneous at presentation: the reasons for revision surgery, the initial diagnosis, and the number of previous surgeries differed between patients. Third, the degree of humeral and glenoid lateralization varied based on the intraoperative status of the patients. Nonetheless measurements were performed to access the amount of lateralization and distalization. Although, the dropout rate is similar to literature for rTSA due to the age and nature of elderly patients, a lost-to-FU of 16% presents another limitation.

The main strength of this work is the unique comparison in revision setting of metallic unipolar and bipolar lateralization in patients with reduced glenoid bone stock. It will be interesting to FU patients for a longer period to determine long-term outcomes, complications, and implant survival rates.

## Conclusion

In our cohort, rTSA presents as a viable option for revision cases and produces good results after failed shoulder arthroplasty, including the infected shoulder. The effect of metallic augmentation on clinical results is not comparable in our cohort to those in literature in primary rTSA setting due to advanced preoperative medialization. However, scapular notching was prevented in all cases with bipolar lateralization.

## Disclaimers:

Funding: Support for this research was provided by Stryker Inc. / Tornier SAS / WrightMedical.

Conflicts of interest: Prof. Scheibel, Dr. Freislederer, and David Endell are consultants for Stryker Corporation. Prof. Moroder is a consultant for Arthrex Inc. The other authors, their immediate families, and any research foundations with which they are affiliated have not received any financial payments or other benefits from any commercial entity related to the subject of this article.

## References

[1] Alentorn-Geli E., Samitier G., Torrens C., Wright T.W. (2015). Reverse shoulder arthroplasty. Part 2: systematic review of reoperations, revisions, problems, and complications. Int J Shoulder Surg.

[2] Antuna S.A., Sperling J.W., Cofield R.H., Rowland C.M. (2001). Glenoid revision surgery after total shoulder arthroplasty. J Shoulder Elbow Surg.

[3] Arenas-Miquelez A., Murphy R.J., Rosa A., Caironi D., Zumstein M.A. (2021). Impact of humeral and glenoid component variations on range of motion in reverse geometry total shoulder arthroplasty: a standardized computer model study. J Shoulder Elbow Surg.

[4] Ascione F., Domos P., Guarrella V., Chelli M., Boileau P., Walch G. (2018). Long-term humeral complications after Grammont-style reverse shoulder arthroplasty. J Shoulder Elbow Surg.

[5] Bacle G., Nové-Josserand L., Garaud P., Walch G. (2017). Long-term outcomes of reverse total shoulder arthroplasty: a follow-up of a previous study. J Bone Joint Surg Am.

[6] Boileau P., Raynier J.L., Chelli M., Gonzalez J.F., Galvin J.W. (2020). Reverse shoulder-allograft prosthesis composite, with or without tendon transfer, for the treatment of severe proximal humeral bone loss. J Shoulder Elbow Surg.

[7] Boileau P., Watkinson D.J., Hatzidakis A.M., Balg F. (2005). Grammont reverse prosthesis: design, rationale, and biomechanics. J Shoulder Elbow Surg.

[8] Bonnevialle N., Melis B., Neyton L., Favard L., Molé D., Walch G. (2013). Aseptic glenoid loosening or failure in total shoulder arthroplasty: revision with glenoid reimplantation. J Shoulder Elbow Surg.

[9] Boutsiadis A., Lenoir H., Denard P.J., Panisset J.-C., Brossard P., Delsol P. (2018). The lateralization and distalization shoulder angles are important determinants of clinical outcomes in reverse shoulder arthroplasty. J Shoulder Elbow Surg.

[10] Cazeneuve J.F., Cristofari D.J. (2011). Long term functional outcome following reverse shoulder arthroplasty in the elderly. Orthop Traumatol Surg Res.

[11] Constant C.R., Gerber C., Emery R.J., Søjbjerg J.O., Gohlke F., Boileau P. (2008). A review of the constant score: modifications and guidelines for its use. J Shoulder Elbow Surg.

[12] Costantini O., Choi D.S., Kontaxis A., Gulotta L.V. (2015). The effects of progressive lateralization of the joint center of rotation of reverse total shoulder implants. J Shoulder Elbow Surg.

[13] Descamps J., Chelli M., Greco V., Azar M., Bessière C., Boileau P. (2023). Subjective shoulder value for sport (SSV-Sport) is a simple, reliable, and valid score to assess shoulder function in athletes. Arthroscopy.

[14] Durchholz H., Salomonsson B., Moroder P., Lambert S., Page R., Audigé L. (2019). Core set of radiographic parameters for shoulder arthroplasty monitoring: criteria defined by an International Delphi Consensus process. JB JS Open Access.

[15] Entezari V., Henry T., Zmistowski B., Sheth M., Nicholson T., Namdari S. (2020). Clinically significant subscapularis failure after anatomic shoulder arthroplasty: is it worth repairing?. J Shoulder Elbow Surg.

[16] Franceschetti E., Ranieri R., Giovanetti de Sanctis E., Palumbo A., Franceschi F. (2020). Clinical results of bony increased-offset reverse shoulder arthroplasty (BIO-RSA) associated with an onlay 145 degrees curved stem in patients with cuff tear arthropathy: a comparative study. J Shoulder Elbow Surg.

[17] Gauci M.O., Cavalier M., Gonzalez J.F., Holzer N., Baring T., Walch G. (2020). Revision of failed shoulder arthroplasty: epidemiology, etiology, and surgical options. J Shoulder Elbow Surg.

[18] Giles J.W., Langohr D.G.G., Johnson J.A., Athwal G.S. (2015). Implant design variations in reverse total shoulder arthroplasty influence the required deltoid force and resultant joint load. Clin Orthop Relat Res.

[19] Giles J.W., Langohr G.D., Johnson J.A., Athwal G.S. (2016). The rotator cuff muscles are antagonists after reverse total shoulder arthroplasty. J Shoulder Elbow Surg.

[20] Gohlke F., Abdelkawi A.A., Eltair H., Aboalata M., Hussein W., Abdrabo M.S. (2020). Revision of failed reverse shoulder arthroplasty—a point of no return?. Obere Extremität.

[21] Gohlke F., Werner B. (2017). [Humeral and glenoid bone loss in shoulder arthroplasty: classification and treatment principles]. Orthopä.

[22] Greiner S., Schmidt C., Herrmann S., Pauly S., Perka C. (2015). Clinical performance of lateralized versus non-lateralized reverse shoulder arthroplasty: a prospective randomized study. J Shoulder Elbow Surg.

[23] Hao K.A., Boschert E.N., O'Keefe D.S., Saengchote S.A., Schoch B.S., Wright J.O. (2023). Comparison of clinical outcomes of revision reverse total shoulder arthroplasty for failed primary anatomic vs. reverse shoulder arthroplasty. JSES Int.

[24] Harris P.A., Taylor R., Thielke R., Payne J., Gonzalez N., Conde J.G. (2009). Research electronic data capture (REDCap)—a metadata-driven methodology and workflow process for providing translational research informatics support. J Biomed Inf.

[25] Heifner J.J., Kumar A.D., Wagner E.R. (2021). Reverse shoulder arthroplasty used for revision of reverse shoulder arthroplasty: a systematic review. JSES Rev Rep Tech.

[26] Henninger H.B., Barg A., Anderson A.E., Bachus K.N., Burks R.T., Tashjian R.Z. (2012). Effect of lateral offset center of rotation in reverse total shoulder arthroplasty: a biomechanical study. J Shoulder Elbow Surg.

[27] Hernandez N.M., Chalmers B.P., Wagner E.R., Sperling J.W., Cofield R.H., Sanchez-Sotelo J. (2017). Revision to reverse total shoulder arthroplasty restores stability for patients with unstable shoulder prostheses. Clin Orthop Relat Res.

[28] Holschen M., Franetzki B., Witt K.A., Liem D., Steinbeck J. (2017). Is reverse total shoulder arthroplasty a feasible treatment option for failed shoulder arthroplasty? A retrospective study of 44 cases with special regards to stemless and stemmed primary implants. Musculoskelet Surg.

[29] Imiolczyk J.P., Audige L., Harzbecker V., Moroder P., Scheibel M. (2022). Metallic humeral and glenoid lateralized implants in reverse shoulder arthroplasty for cuff tear arthropathy and primary osteoarthritis. JSES Int.

[30] Imiolczyk J.P., Imiolczyk T., Goralczyk A., Scheibel M., Freislederer F. (2024). Lateralization and distalization shoulder angles do not predict outcomes in reverse shoulder arthroplasty for cuff tear arthropathy. J Shoulder Elbow Surg.

[31] Kim S.H., Wise B.L., Zhang Y., Szabo R.M. (2011). Increasing incidence of shoulder arthroplasty in the United States. JBJS.

[32] Langohr G.D., Giles J.W., Athwal G.S., Johnson J.A. (2015). The effect of glenosphere diameter in reverse shoulder arthroplasty on muscle force, joint load, and range of motion. J Shoulder Elbow Surg.

[33] Malawer M.M. (1991). Tumors of the shoulder girdle. Technique of resection and description of a surgical classification. Orthop Clin North Am.

[34] Marzel A., Schwyzer H.K., Kolling C., Moro F., Flury M., Glanzmann M.C. (2020). The Schulthess local shoulder arthroplasty Registry (SAR): cohort profile. BMJ Open.

[35] Matassi F., Botti A., Sirleo L., Carulli C., Innocenti M. (2013). Porous metal for orthopedics implants. Clin Cases Miner Bone Metab.

[36] Melis B., Bonnevialle N., Neyton L., Lévigne C., Favard L., Walch G. (2012). Glenoid loosening and failure in anatomical total shoulder arthroplasty: is revision with a reverse shoulder arthroplasty a reliable option?. J Shoulder Elbow Surg.

[37] Merolla G., Tartarone A., Sperling J.W., Paladini P., Fabbri E., Porcellini G. (2017). Early clinical and radiological outcomes of reverse shoulder arthroplasty with an eccentric all-polyethylene glenosphere to treat failed hemiarthroplasty and the sequelae of proximal humeral fractures. Int Orthop.

[38] Nunes B., Linhares D., Costa F., Neves N., Claro R., Silva M.R. (2021). Lateralized versus nonlateralized glenospheres in reverse shoulder arthroplasty: a systematic review with meta-analysis. J Shoulder Elbow Surg.

[39] Raiss P., Neumann R. (2020). Bipolar lateralization in reverse shoulder arthroplasty for avoidance of scapular notching. Obere Extremität.

[40] Ravi V., Murphy R., Moverley R., Derias M., Phadnis J. (2021). 654 outcome and complications following revision shoulder arthroplasty. A systematic review and meta-analysis. Br J Surg.

[41] Reddy A.K., Checketts J.X., Stephens B.J., Anderson J.M., Cooper C.M., Hunt T. (2022). Complication and revision rates after reverse total shoulder revision from hemiarthroplasty: a systematic review. Shoulder Elbow.

[42] Routman H.D., Flurin P.H., Wright T.W., Zuckerman J.D., Hamilton M.A., Roche C.P. (2015). Reverse shoulder arthroplasty prosthesis design classification system. Bull Hosp Jt Dis.

[43] Sanchez-Sotelo J., Wagner E.R., Sim F.H., Houdek M.T. (2017). Allograft-prosthetic composite Reconstruction for massive proximal humeral bone loss in reverse shoulder arthroplasty. J Bone Joint Surg Am.

[44] Sheth M.M., Sholder D., Getz C.L., Williams G.R., Namdari S. (2019). Revision of failed hemiarthroplasty and anatomic total shoulder arthroplasty to reverse total shoulder arthroplasty. J Shoulder Elbow Surg.

[45] Sirveaux F., Favard L., Oudet D., Huquet D., Walch G., Molé D. (2004). Grammont inverted total shoulder arthroplasty in the treatment of glenohumeral osteoarthritis with massive rupture of the cuff. Results of a multicentre study of 80 shoulders. J Bone Joint Surg Br.

[46] Southam B.R., Bedeir Y.H., Johnson B.M., Hasselfeld K.A., Kloby M.A., Grawe B.M. (2023). Clinical and radiological outcomes in lateralized versus nonlateralized and distalized glenospheres in reverse total shoulder arthroplasty: a randomized control trial. J Shoulder Elbow Surg.

[47] Werner B.C., Lederman E., Gobezie R., Denard P.J. (2021). Glenoid lateralization influences active internal rotation after reverse shoulder arthroplasty. J Shoulder Elbow Surg.

[48] Werner B.S., Chaoui J., Walch G. (2017). The influence of humeral neck shaft angle and glenoid lateralization on range of motion in reverse shoulder arthroplasty. J Shoulder Elbow Surg.

[49] Werner B.S., Chaoui J., Walch G. (2017). The influence of humeral neck shaft angle and glenoid lateralization on range of motion in reverse shoulder arthroplasty. J Shoulder Elbow Surg.

[50] Werthel J.D., Walch G., Vegehan E., Deransart P., Sanchez-Sotelo J., Valenti P. (2019). Lateralization in reverse shoulder arthroplasty: a descriptive analysis of different implants in current practice. Int Orthop.

