# Supplementary material for: Clinical and radiological results of reverse total shoulder arthroplasty with or without lateralization as revision procedure for failed arthroplasty
Source: JSES Int. 2024 Nov 27;9(2):477–85. doi: 10.1016/j.jseint.2024.10.014 (PMC11962622; doi:10.1016/j.jseint.2024.10.014)
Supplement: Supplementary File 1 [file mmc1.docx]

**Supplementary file 1**

**Article title** Clinical and radiological results of reverse shoulder arthroplasty with or without lateralization as revision procedure for failed arthroplasty

**Journal name** Journal of Shoulder and Elbow Surgery

**Author names** Jan-Philipp Imiolczyk, MD; Laurent Audigé, DVM, PhD; Florian Freislederer, MD; Philipp Moroder, MD, Prof; David Endell, MD; Raphael Trefzer, MD; Markus Scheibel, Prof, MD

**Affiliation** Schulthess Klinik, CH-8008 Zurich, Switzerland

**E-mail address** markus.scheibel@kws.ch

**Change of shoulder range of motion (ROM) parameters, strength and functional scores per study group**

**Group non-latRSA = reverse shoulder arthroplasty (RSA) with no baseplate offset with a Grammont-type 155° stem**

|  | **Baseline** | | **Final FU** | |  |  |
| --- | --- | --- | --- | --- | --- | --- |
| Outcome parameters | n(%) | mean (SD) | n(%) | mean (SD) | Change (95% CI) | P-value |
| Flexion (°) | 12 | 50 (31) | 10 | 135 (32) | 88 (64 to 111) | 0.005 |
| Abduction (°) | 12 | 49 (29) | 10 | 121 (36) | 73 (55 to 90) | 0.005 |
| External rotation in 0° abduction (°) | 12 | 17 (26) | 8 | 31 (25) | 9 (-1 to 19) | 0.103 |
| Internal rotation (Apley's test) |  |  |  |  |  | 1.000 |
| Lateral thigh | 5 (42) |  | 2 (22) |  |  |  |
| Buttock | 1 (8) |  | 1 (11) |  |  |  |
| Lumbosacral region | 2 (17) |  | 4 (44) |  |  |  |
| Waist (L3) | 3 (25) |  | 2 (22) |  |  |  |
| T12 vertebra | 1 (8) |  | - |  |  |  |
| Interscapular T7 | - |  | - |  |  |  |
| Strength in abduction (kg) | 12 | 0.9 (2.1) | 10 | 3.9 (3.9) | 2.8 (1.2 to 4.5) | 0.011 |
| Subjective Shoulder Value (%) | 12 | 25 (13) | 12 | 73 (16) | 48 (40 to 55) | 0.002 |
| Pain level NRS (0-10=max) | 12 | 5.9 (3.2) | 12 | 1.8 (2.6) | -4.1 (-5.9 to -2.4) | 0.005 |
| Constant Murley Score (0=min 100=max) | 12 | 23 (16) | 9 | 62 (15) | 39 (28 to 50) | 0.008 |

SD = standard deviation; 95% CI = 95% Confidence Interval; P-value = Wilcoxon signed-rank test p-value; NRS = Numeric Rating Scale

**Group latRSA = lateralized RSA with metallic baseplate augmentation**

|  | **Baseline** | | **Final FU** |  |  |  |
| --- | --- | --- | --- | --- | --- | --- |
| Outcome parameters | n(%) | mean (SD) | n(%) | mean (SD) | Change (95% CI) | P-value |
| Flexion (°) | 15 | 59 (38) | 15 | 134 (37) | 75 (53 to 96) | 0.001 |
| Abduction (°) | 15 | 52 (36) | 15 | 121 (41) | 69 (45 to 93) | 0.001 |
| External rotation in 0° abduction (°) | 15 | 11 (16) | 15 | 21 (20) | 10 (2 to 18) | 0.026 |
| Internal rotation (Apley's test) |  |  |  |  |  | 0.250 |
| Lateral thigh | 6 (40) |  | 1 (7) |  |  |  |
| Buttock | 5 (33) |  | 7 (47) |  |  |  |
| Lumbosacral region | 4 (27) |  | 4 (27) |  |  |  |
| Waist (L3) | - |  | 1 (7) |  |  |  |
| T12 vertebra | - |  | 1 (7) |  |  |  |
| Interscapular T7 | - |  | 1 (7) |  |  |  |
| Strength in abduction (kg) | 15 | 0.9 (2.4) | 15 | 3.2 (2.4) | 2.3 (1.1 to 3.5) | 0.005 |
| Subjective Shoulder Value (%) | 15 | 23 (23) | 15 | 67 (23) | 44 (32 to 55) | 0.001 |
| Pain level NRS (0-10=max) | 15 | 7.0 (2.9) | 15 | 1.5 (1.7) | -5.5 (-6.7 to -4.2) | 0.001 |
| Constant Murley Score (0=min 100=max) | 15 | 21 (20) | 15 | 60 (18) | 38 (29 to 47) | 0.001 |

SD = standard deviation; 95% CI = 95% Confidence Interval; P-value = Wilcoxon signed-rank test p-value; NRS = Numeric Rating Scale

**Group bi-latRSA = lateralized RSA with metallic baseplate augmentation and additional humeral lateralization using a 145° onlay curved stem**

|  | **Baseline** | | **Final FU** |  |  |  |
| --- | --- | --- | --- | --- | --- | --- |
| Outcome parameters | n(%) | mean (SD) | n(%) | mean (SD) | Change (95% CI) | P-value |
| Flexion (°) | 11 | 71 (37) | 11 | 135 (35) | 64 (40 to 87) | 0.004 |
| Abduction (°) | 11 | 73 (31) | 11 | 136 (33) | 63 (42 to 84) | 0.004 |
| External rotation in 0° abduction (°) | 11 | 22 (20) | 11 | 37 (20) | 15 (3 to 27) | 0.045 |
| Internal rotation (Apley's test) |  |  |  |  |  | 0.125 |
| Lateral thigh | 2 (18) |  | - |  |  |  |
| Buttock | 4 (36) |  | 4 (36) |  |  |  |
| Lumbosacral region | 4 (36) |  | 1 (9) |  |  |  |
| Waist (L3) | - |  | 5 (45) |  |  |  |
| T12 vertebra | 1 (9) |  | - |  |  |  |
| Interscapular T7 | - |  | 1 (9) |  |  |  |
| Strength in abduction (kg) | 11 | 0.6 (1.1) | 11 | 4.3 (2.5) | 3.6 (2.2 to 5.1) | 0.005 |
| Subjective Shoulder Value (%) | 11 | 27 (20) | 11 | 62 (20) | 35 (23 to 46) | 0.004 |
| Pain level NRS (0-10=max) | 11 | 5.3 (2.8) | 11 | 2.6 (2.3) | -2.7 (-3.9 to -1.4) | 0.006 |
| Constant Murley Score (0=min 100=max) | 11 | 28 (15) | 11 | 62 (20) | 34 (23 to 45) | 0.004 |

SD = standard deviation; 95% CI = 95% Confidence Interval; P-value = Wilcoxon signed-rank test p-value; NRS = Numeric Rating Scale
