# Supplementary material for: Clinical and radiological results of reverse total shoulder arthroplasty with or without lateralization as revision procedure for failed arthroplasty
Source: JSES Int. 2024 Nov 27;9(2):477–85. doi: 10.1016/j.jseint.2024.10.014 (PMC11962622; doi:10.1016/j.jseint.2024.10.014)
Supplement: Supplementary File 2 [file mmc2.docx]

**Supplementary file 2**

**Article title** Clinical and radiological results of reverse shoulder arthroplasty with or without lateralization as revision procedure for failed arthroplasty

**Journal name** Journal of Shoulder and Elbow Surgery

**Author names** Jan-Philipp Imiolczyk, MD; Laurent Audigé, DVM, PhD; Florian Freislederer, MD; Philipp Moroder, MD, Prof; David Endell, MD; Raphael Trefzer, MD; Markus Scheibel, Prof, MD

**Affiliation** Schulthess Klinik, CH-8008 Zurich, Switzerland

**E-mail address** markus.scheibel@kws.ch

**Baseline and postoperative shoulder range of motion (ROM) parameters, strength and functional scores**

|  | **non-latRSA** |  | **latRSA** |  | **bi-latRSA** |  | Adjusted | Model |
| --- | --- | --- | --- | --- | --- | --- | --- | --- |
| Active range of motion parameters | n | mean (SD) | n | mean (SD) | n | mean (SD) | p-value | p-value* |
| Flexion (°) |  |  |  |  |  |  |  | 0.982 |
| Baseline | 12 | 50 (31) | 15 | 59 (38) | 11 | 71 (37) |  |  |
| 12 months | 8 | 131 (27) | 14 | 123 (41) | 8 | 144 (31) | 0.451 |  |
| 24 months | 10 | 135 (32) | 15 | 134 (37) | 11 | 135 (35) | 0.854 |  |
| Abduction (°) |  |  |  |  |  |  |  | 0.722 |
| Baseline | 12 | 49 (29) | 15 | 52 (36) | 11 | 73 (31) |  |  |
| 12 months | 8 | 126 (35) | 14 | 108 (40) | 8 | 138 (25) | 0.273 |  |
| 24 months | 10 | 121 (36) | 15 | 121 (41) | 11 | 136 (33) | 0.923 |  |
| External rotation in 0° abduction (°) |  |  |  |  |  |  |  | 0.373 |
| Baseline | 12 | 17 (26) | 15 | 11 (16) | 11 | 22 (20) |  |  |
| 12 months | 8 | 40 (24) | 14 | 18 (18) | 8 | 31 (13) | 0.311 |  |
| 24 months | 8 | 31 (25) | 15 | 21 (20) | 11 | 37 (20) | 0.527 |  |
|  |  |  |  |  |  |  |  |  |

**Continued**

|  | **non-latRSA** |  | **latRSA** |  | **bi-latRSA** |  | Adjusted | Model |
| --- | --- | --- | --- | --- | --- | --- | --- | --- |
| Strength and functional scores | n | mean (SD) | n | mean (SD) | n | mean (SD) | p-value | p-value |
| Strength in abduction (kg) |  |  |  |  |  |  |  | 0.691 |
| Baseline | 12 | 0.9 (2.1) | 15 | 0.9 (2.4) | 11 | 0.6 (1.1) |  |  |
| 12 months | 8 | 5.1 (3.8) | 14 | 3.2 (3.1) | 8 | 4.4 (2.6) | 0.612 |  |
| 24 months | 10 | 3.9 (3.9) | 15 | 3.2 (2.4) | 11 | 4.3 (2.5) | 0.741 |  |
| Subjective Shoulder Value (0=worst, 100=best) |  |  |  |  |  |  |  | 0.320 |
| Baseline | 12 | 25 (13) | 15 | 23 (23) | 11 | 27 (20) |  |  |
| 12 months | 8 | 73 (14) | 13 | 58 (27) | 8 | 69 (22) | 0.268 |  |
| 24 months | 12 | 73 (16) | 15 | 67 (23) | 11 | 62 (20) | 0.222 |  |
| Pain NRS (0=no pain, 10=maximum pain) |  |  |  |  |  |  |  | 0.136 |
| Baseline | 12 | 5.9 (3.2) | 15 | 7.0 (2.9) | 11 | 5.3 (2.8) |  |  |
| 12 months | 8 | 1.2 (2.1) | 13 | 1.4 (1.6) | 8 | 1.9 (1.9) | 0.755 |  |
| 24 months | 12 | 1.8 (2.6) | 15 | 1.5 (1.7) | 11 | 2.6 (2.3) | 0.040 |  |
| Constant Murley Score (0-100=best) |  |  |  |  |  |  |  | 0.929 |
| Baseline | 12 | 23 (16) | 15 | 21 (20) | 11 | 28 (15) |  |  |
| 12 months | 7 | 63 (15) | 13 | 55 (18) | 8 | 66 (16) | 0.509 |  |
| 24 months | 9 | 62 (15) | 15 | 60 (18) | 11 | 62 (20) | 0.887 |  |
|  |  |  |  |  |  |  |  |  |

Group non-latRSA = reverse shoulder arthroplasty (RSA) with no baseplate offset with a Grammont-type 155° stem; Group latRSA = lateralized RSA with metallic baseplate augmentation; Group bi-latRSA = lateralized RSA with metallic baseplate augmentation and additional humeral lateralization using a 145° onlay curved stem

SD = standard deviation; NRS = Numeric Rating Scale
* p-value = model p-value for group effect adjusted for age, gender and baseline preoperative values (Adjusted: separately at 12 and 24-month follow-up; Model: Mixed model including both followup time-points)
