# Supplementary material for: Clinical and radiological results of reverse total shoulder arthroplasty with or without lateralization as revision procedure for failed arthroplasty
Source: JSES Int. 2024 Nov 27;9(2):477–85. doi: 10.1016/j.jseint.2024.10.014 (PMC11962622; doi:10.1016/j.jseint.2024.10.014)
Supplement: Supplementary File 3 [file mmc3.docx]

**Supplementary file 3**

**Article title** Clinical and radiological results of reverse shoulder arthroplasty with or without lateralization as revision procedure for failed arthroplasty

**Journal name** Journal of Shoulder and Elbow Surgery

**Author names** Jan-Philipp Imiolczyk, MD; Laurent Audigé, DVM, PhD; Florian Freislederer, MD; Philipp Moroder, MD, Prof; David Endell, MD; Raphael Trefzer, MD; Markus Scheibel, Prof, MD

**Affiliation** Schulthess Klinik, CH-8008 Zurich, Switzerland

**E-mail address** markus.scheibel@kws.ch

**Change of shoulder range of motion (ROM) parameters, strength and functional scores per aseptic vs septic case subgroup**

**Aseptic revision cases**

|  | **Baseline** | | **Final follow-up** | |  |  |
| --- | --- | --- | --- | --- | --- | --- |
| Outcome parameters | n(%) | mean (SD) | n(%) | mean (SD) | Change (95% CI) | P-value |
| Flexion (°) | 21 | 70 (36) | 19 | 129 (35) | 59 (45 to 74) | <0.001 |
| Abduction (°) | 21 | 70 (33) | 19 | 127 (35) | 55 (45 to 65) | <0.001 |
| External rotation in 0° abduction (°) | 21 | 24 (22) | 18 | 31 (22) | 5 (-2 to 13) | 0.171 |
| Internal rotation (Apley's test) |  |  |  |  |  | 0.088 |
| Lateral thigh | 4 (19) |  | - |  |  |  |
| Buttock | 5 (24) |  | 7 (39) |  |  |  |
| Lumbosacral region | 7 (33) |  | 4 (22) |  |  |  |
| Waist (L3) | 3 (14) |  | 7 (39) |  |  |  |
| T12 vertebra | 2 (10) |  | - |  |  |  |
| Interscapular T7 | - |  | - |  |  |  |
| Strength in abduction (kg) | 21 | 1.4 (2.5) | 19 | 4.2 (3.2) | 2.7 (1.6 to 3.8) | <0.001 |
| Subjective Shoulder Value (%) | 21 | 33 (22) | 21 | 70 (21) | 37 (28 to 46) | <0.001 |
| Pain level NRS (0-10=max) | 21 | 5.2 (3.0) | 21 | 2.2 (2.6) | -3.0 (-4.2 to -1.8) | <0.001 |
| Constant Murley Score (0=min 100=max) | 21 | 31 (18) | 18 | 63 (19) | 30 (22 to 37) | <0.001 |

**Septic revision cases**

|  | **Baseline** | | **Final follow-up** | |  |  |
| --- | --- | --- | --- | --- | --- | --- |
| Outcome parameters | n(%) | mean (SD) | n(%) | mean (SD) | Change (95% CI) | P-value |
| Flexion (°) | 17 | 48 (33) | 17 | 140 (33) | 92 (72 to 113) | <0.001 |
| Abduction (°) | 17 | 42 (29) | 17 | 125 (40) | 83 (60 to 105) | <0.001 |
| External rotation in 0° abduction (°) | 17 | 6 (14) | 16 | 25 (21) | 18 (10 to 26) | 0.001 |
| Internal rotation (Apley's test) |  |  |  |  |  | 0.189 |
| Lateral thigh | 9 (53) |  | 3 (18) |  |  |  |
| Buttock | 5 (29) |  | 5 (29) |  |  |  |
| Lumbosacral region | 3 (18) |  | 5 (29) |  |  |  |
| Waist (L3) | - |  | 1 (6) |  |  |  |
| T12 vertebra | - |  | 1 (6) |  |  |  |
| Interscapular T7 | - |  | 2 (12) |  |  |  |
| Strength in abduction (kg) | 17 | 0.1 (0.4) | 17 | 3.2 (2.5) | 3.1 (1.8 to 4.3) | <0.001 |
| Subjective Shoulder Value (%) | 17 | 16 (8) | 17 | 64 (19) | 49 (40 to 57) | <0.001 |
| Pain level NRS (0-10=max) | 17 | 7.3 (2.6) | 17 | 1.6 (1.5) | -5.7 (-6.7 to -4.7) | <0.001 |
| Constant Murley Score (0=min 100=max) | 17 | 15 (10) | 17 | 59 (16) | 45 (38 to 52) | <0.001 |

SD = standard deviation; 95% CI = 95% Confidence Interval; P-value = Wilcoxon signed-rank test p-value; NRS = Numeric Rating Scale
